# Right Structural and Functional Reorganization in Four-Year-Old Children with Perinatal Arterial Ischemic Stroke Predict Language Production

**DOI:** 10.1523/ENEURO.0447-18.2019

**Published:** 2019-08-28

**Authors:** Clément François, Pablo Ripollés, Laura Ferreri, Jordi Muchart, Joanna Sierpowska, Carme Fons, Jorgina Solé, Monica Rebollo, Robert J. Zatorre, Alfredo Garcia-Alix, Laura Bosch, Antoni Rodriguez-Fornells

**Affiliations:** 1 Cognition and Brain Plasticity Group, Bellvitge Biomedical Research Institute (IDIBELL), L’Hospitalet de Llobregat, Barcelona 08907, Spain; 2 Department of Cognition, Development and Educational Science, University of Barcelona, Barcelona 08035, Spain; 3 Poeppel Lab, Department of Psychology, New York University, NY 10003; 4 Institut de Recerca Sant Joan de Déu. Hospital Sant Joan de Déu, Universitat de Barcelona, Barcelona 08950, Spain; 5 Donders Institute for Brain, Cognition and Behaviour, Radboud University, 6525 HR, Nijmegen, The Netherlands; 6 Department of Medical Psychology, Radboud University Medical Center, Nijmegen, The Netherlands; 7 Service of Genetic and Molecular Medicine, Hospital Sant Joan de Déu, Barcelona 08950, Spain; 8 Cognitive Neuroscience Unit, Montreal Neurological Institute, McGill University, Montreal H3A 2B4, Canada; 9 Centro de Investigación Biomédica en Red de Enfermedades Raras (CIBERER), U724, Madrid, Spain; 10 Institute of Neuroscience (UBNeuro), University of Barcelona, Barcelona, 08035, Spain; 11 Catalan Institution for Research and Advanced Studies (ICREA), Barcelona, 08010, Spain

**Keywords:** diffusion tensor imaging, fMRI, hyperconnectivity, interhemispheric plasticity, language production, perinatal arterial ischemic stroke

## Abstract

Brain imaging methods have contributed to shed light on the mechanisms of recovery after early brain insult. The assumption that the unaffected right hemisphere can take over language functions after left perinatal stroke is still under debate. Here, we report how patterns of brain structural and functional reorganization were associated with language outcomes in a group of four-year-old children with left perinatal arterial ischemic stroke (PAIS). Specifically, we gathered specific fine-grained developmental measures of receptive and productive aspects of language as well as standardized measures of cognitive development. We also collected structural neuroimaging data as well as functional activations during a passive listening story-telling fMRI task and a resting state session (rs-fMRI). Children with a left perinatal stroke showed larger lateralization indices of both structural and functional connectivity of the dorsal language pathway towards the right hemisphere that, in turn, were associated with better language outcomes. Importantly, the pattern of structural asymmetry was significantly more right-lateralized in children with a left perinatal brain insult than in a group of matched healthy controls. These results strongly suggest that early lesions of the left dorsal pathway and the associated perisylvian regions can induce the interhemispheric transfer of language functions to right homolog regions. This study provides combined evidence of structural and functional brain reorganization of language networks after early stroke with strong implications for neurobiological models of language development.

## Significance Statement

The prevalent theories explaining the functional recovery of language functions after perinatal ischemic stroke strikingly differ on the role of perilesional functionally spared regions as opposed to the homologous non-affected contralesional brain areas. Here, we assessed how patterns of brain functional and structural reorganization were associated with language outcomes in a group of four-year-old children with left perinatal arterial ischemic stroke (PAIS). Larger lateralization indices of both functional and structural connectivity toward the right hemisphere were associated with higher levels of language development. Thus, interhemispheric plasticity through structural and functional hyperconnectivity mechanisms might be crucial in early damage, probably through the degeneration of neurons projecting from temporal to frontal areas together with contralateral axonal sprouting over the right hemisphere.

## Introduction

In adults, language processing relies on a largely distributed network mainly involving perisylvian brain regions in the left hemisphere (Friederici, 2011; Price, 2012). These brain areas are interconnected through white matter (WM) fiber bundles, altogether forming the dorsal and ventral pathways involved in language processing (Catani et al., 2005; Hickok and Poeppel, 2007; Rauschecker and Scott, 2009). According to the dual-stream models, the dorsal pathway includes the arcuate fasciculus (AF) composed of the long segment that connects superior posterior temporal regions with Broca’s area, the anterior segment connecting the inferior parietal lobe (IPL) with Broca’s area, and the posterior segment from pST regions to the IPL (Catani et al., 2005, 2007). Functionally, the AF is known to contribute to sound-to-articulation transformations (Liberman and Mattingly, 1985; Hickok and Poeppel, 2007), phonological word-learning (López-Barroso et al., 2013), audio-motor integration (Assaneo et al., 2019), phonological processing (Vandermosten et al., 2012), and working memory (Meyer et al., 2014). The ventral stream connects the occipital superior temporal, angular gyrus and inferior frontal gyri and is formed by the inferior fronto-occipital fasciculus (IFOF), inferior longitudinal fasciculus (ILF), and uncinate fasciculus (UF; Bajada et al., 2015). This ventral pathway rather contributes to semantic processing, sound-to-meaning mapping, semantic word-learning and auditory object recognition (Catani et al., 2003; Saur et al., 2008; Friederici, 2009; Ripollés et al., 2017). Interestingly, neuroimaging studies have revealed that newborns may recruit a bilateral neural network with an already left predominance for linguistic stimuli (Shultz et al., 2014; Dehaene-Lambertz and Spelke, 2015). Although it is still under debate, some of the white-matter tracts such as the AFs are already developed and functional at birth, while others such as the UF are still developing due to a delayed maturation (Zhang et al., 2007; Perani et al., 2011; Brauer et al., 2013; Bajada et al., 2015). Despite the fact that low-level language functions may already be present at birth, as reflected by left cortical activations for specific spectro-temporal features of the speech input (Dehaene-Lambertz et al., 2002, 2006; Telkemeyer et al., 2009; Perani et al., 2010), the later development of higher-order language functions may involve a graded lateralization toward the left hemisphere emerging with age (Brown et al., 2005; Szaflarski et al., 2006). For instance, the cortical maturation of temporoparietal regions has been shown to occur much slower than the maturation of frontal brain regions (Leroy et al., 2011). This slow maturation has also been evidenced at the WM level with a slower maturation and higher sensitivity to environmental factors of the right posterior segment of the AF as compared to the anterior and long segments (Budisavljevic et al., 2015). These results favor a plastic and experience-dependent view of the neural bases of language acquisition in typically developing children.

In this context, the study of young children with early brain lesions such as perinatal arterial ischemic stroke (PAIS) provide a unique opportunity to study brain reorganization and plasticity mechanisms taking place before language development unfolds. PAIS occurs in one in 4000 births (Nelson and Lynch, 2004) and may have long-term negative effects on children’s cognitive development and language functions compared to typically developing peers (Ballantyne et al., 2008; Westmacott et al., 2009; Westmacott et al., 2010; Reilly, et al., 2013). Nonetheless, as opposed to stroke occurring during adulthood, PAIS over the left hemisphere does not generally induce post-stroke aphasia (Bates et al., 2001; Stiles et al., 2005) although very recent data suggest the possible existence of a developmental form of conduction aphasia after neonatal stroke (Northam et al., 2018; Schlaug, 2018). Due to the low incidence of PAIS, no previous neuroimaging studies have been conducted in this population during the pre-school period when learning and neuroplastic processes are taking place. Here, we assessed for the first time how patterns of brain structural and functional reorganization were associated with language outcomes in a group of four-year-old children with left PAIS. To this aim, we gathered fine-grained measures of receptive and productive aspects of language as well as standardized measures of cognitive development. We also collected both structural and functional neuroimaging data under light anesthesia. Importantly, we used a group of healthy children from the Pediatric MRI Data Repository [National Institutes of Health (NIH) MRI Study of Normal Brain Development) matched in age and gender to serve as a control for the structural connectivity analyses.

Considering the role of perilesional functionally spared regions as opposed to the homologous non-affected contralesional brain areas in the current theories of functional recovery after early stroke, we expected children with perinatal stroke affecting the left AF to be the most impaired in language functions. This pattern of results would confirm that the functional integrity of the left hemisphere is mandatory to achieve normal language development (Raja-Beharelle et al., 2010). However, if the right hemisphere is able to “take over” language functions usually supported by the left hemisphere (Staudt et al., 2002; Tillema, et al., 2008; Tivarus et al., 2012), then we should observe a significant relationship between structural parameters (i.e., microstructural properties of language-related WM pathways) and/or functional activations in the right hemisphere and measures of receptive and expressive language, in line with previous evidence from adults on the role of the right dorsal pathway in recovery from left stroke-induced aphasia (Forkel et al., 2014; Pani et al., 2016).

## Materials and Methods

### Ethics and participants

Six children with symptomatic PAIS (two girls) over the left hemisphere took part in the present study. These children were part of a larger prospective observational multicenter study about acute symptomatic PAIS. The selection of these children was made based on the minimum age at which the behavioural and neuroimaging evaluations could be done at a reasonable time interval for all of them. All but two children received speech therapy on a weekly basis (L2 and L6). Symptomatic PAIS was defined as the finding of an ischemic lesion by MRI study in the territory of the main cerebral arteries [middle cerebral artery (MCA), anterior cerebral artery (ACA), and posterior cerebral artery (PCA)] in an infant presenting with seizures, recurrent apnea or acute neurologic deficit during the first days of life. Infants with (1) clear sign of tissue loss or atrophy in MRI suggestive of an old infarction that occurred before birth; (2) vascular malformation; (3) congenital or chromosomic anomalies; (4) metabolic or infectious diseases; (5) complex heart congenital diseases or infants with extra corporeal membrane oxygenation, were excluded from the study. Specifically, MRI-based diagnostics were performed on anatomic data acquired on a 1.5 T whole-body MRI scanner with a specific neonatal head coil within 7 d after the occurrence of the neurologic symptoms. These MRI data allowed classifying the lesions according to the arterial territory involved (i.e., four segments: M1–M4). The classification of each infarct was obtained by two independent neurologists blind to the clinical data. Discrepancies in the classification of the PAIS were discussed and solved by consensus at joint revaluation among three observers: AGA and two neurologists blind to the clinical data. Motor impairment was considered when infants had clear clinical signs of monoplegia/hemiplegia (GMFCS at least I and/or BFMF at I level). Infants with minor abnormalities such as precocious handedness, mild abnormalities of tone or reflexes that did not interfere with functions were not considered to have a motor impairment. Importantly, while all children presented with seizures during the three first days following birth, no children suffered from epilepsy at the time of the MRI scan

**Figure 1.**
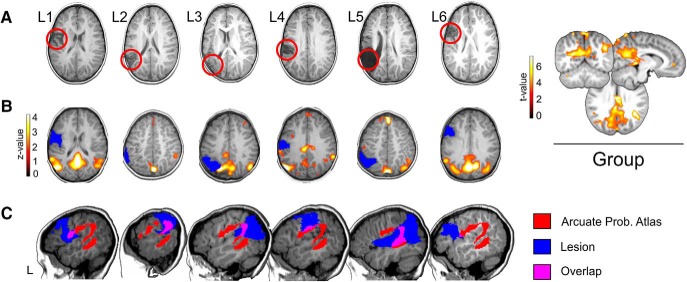
***A***, Depiction of the structural lesions in native space using a T1-w axial image. The lesions can be seen as hypo-intense areas and are highlighted with a red circle. Neurologic convention is used. ***B***, Individual DMNs for each patient, obtained using group spatial ICA on the resting state data (see Materials and Methods). Individual data are in z-scores (*p* < 0.05, uncorrected) and presented over each patient’s T1 registered to a common space (four-year-old MRI brain template obtained from the Neurodevelopmental MRI Database; see Materials and Methods). This was done as a demonstration of data quality, given that propofol can reduce connectivity in cortical areas of significance for the current study (Boveroux et al., 2010). On the right, the average DMN for all patients is depicted, using *t* values (*p* < 0.01, uncorrected) over a canonical four-year-old MRI brain template. ***C***, Probability templates for the left arcuate (anterior, posterior, and long segment) were extracted from the Tractotron atlas (Thiebaut de Schotten et al., 2011; Rojkova et al., 2016), thresholded at a 80% (only voxels having a 80% probability of being part of the AF according to the atlas are shown) and registered to a common space (the four-year-old MRI brain template obtained from the Neurodevelopmental MRI Database). For each patient, here we depict the arcuate probabilistic atlas (in red), the lesion (in blue), and the overlap between them (in pink) over a sagittal slice of the left hemisphere. Neurologic convention is used. L, left hemisphere.

Brain MRI was also acquired at 3.8 years (SD = 0.26) to control for the residual lesion and to obtain the functional and structural data reported here. These MRI data demonstrated that all children had infarctions of the MCA affecting several brain regions (Table 1; Fig. 1). At the time of the study, all the children showed a right-hand preference. The parents of the children received information on the study in written and spoken form and their written consent was obtained before the study begun. The study was approved by the Ethics Committee of a location which will be identified if the article is published in accordance with the Declaration of Helsinki.

**Table 1.** Children demographic data and lesions main features

Patientcode	Age at scan(years)	Gender	Gestational age at birth(weeks)	Birth weight(g)	Clinical debut(hours of age)	Age of MRI diagnostic (d)	Vascularterritory	Stroke volume at birth (ml)	Motor impairment(hemiplegia)	Epilepsy^a^
L1	4	M	41	3160	Clonic seizures at 12 h	4	M2 L	18890	No	No
L2	3.5	F	41	2960	Clonic seizures at 26 h	10	M4 L	47428	Yes	No
L3	4	M	40	3560	Clonic seizures at 48 h	20	M1 Post-bifurcation L	17588	No	No
L4	4	F	41	2600	Clonic seizures at 18 h	4	M2 sup L	27882	No	No
L5	4	M	39	3340	Clonic seizures at 24 h	5	M1 Post-bifurcation L	36512	No	No
L6	3.5	M	40	3025	Clonic seizures at 41 h	5	M1 Post-bifurcation L	23509	Yes	No

a Epilepsy = at least two recurrent and unprovoked seizures.

In addition, we used a group of nine healthy children (three females, 4.30 ± 1.09 years of age) from an MRI pediatric repository to serve as a control for the structural connectivity analyses. The control group and the patients were matched in age [*t*_(13)_ = 1.02; *p* = 0.32. No difference in gender was found (χ^2^ = 0.313; *p* = 0.57). In particular, data used in the preparation of this article were obtained from the Pediatric MRI Data Repository created by the NIH MRI Study of Normal Brain Development (we searched for all children data between three and six years old having DW-MRI data). This is a multi-site, longitudinal study of typically developing children, from ages newborn through young adulthood, conducted by the Brain Development Cooperative Group and supported by the National Institute of Child Health and Human Development, the National Institute on Drug Abuse, the National Institute of Mental Health, and the National Institute of Neurologic Disorders and Stroke (contracts N01-HD02-3343, N01-MH9-0002, and N01-NS-9-2314, N01-NS-9-2315, N01-NS-9-2316, N01-NS-9-2317, N01-NS-9-2319, and N01-NS-9-2320). A listing of the participating sites and a complete listing of the study investigators can be found at https://www.bic.mni.mcgill.ca/nihpd_info/info2/participating_centers.html. This manuscript reflects the views of the authors and may not reflect the opinions or views of the NIH.

### Neuropsychological assessment

#### Standard neuropsychological testing

Cognitive functions in the domains of memory and learning, visuospatial processing, sensorimotor skills, and language were assessed using the NEPSY-II battery with the specific subtest suitable to test three- to four-year-old children (Korkman et al., 2007, Spanish adaptation). Specifically, we assessed: (1) language functions (subtests: comprehension of instructions; body part naming and identification; words generation; phonological processing; and speeded naming); (2) memory and learning functions (subtests: memory for designs; narrative memory; and sentence repetition); (3) visuospatial processing (subtests: block construction; and design copying); and (4) sensorimotor processing (subtests: imitating hand positions and, visuomotor precision). Raw scores for each subtest were converted into scalar values and compared to normative values (mean = 10, SD = 2).

#### Language measures

Receptive vocabulary was assessed using the Peabody picture vocabulary test (PPVT-III; Dunn et al., 1997, with Spanish norms) which offers a raw score that can be transformed into a percentile rank, a receptive verbal IQ (mean = 100, SD = 15) and the corresponding developmental age.

Phonological development assessed phonological production of 32 common words, mostly disyllabic, involving 15 different word shapes and covering all consonantal segments and segment combinations in Spanish (Bosch, 2004). This assessment tool uses pictures favoring utterance production rather than simple naming. From children’s spontaneous production, phonological mean length of utterance (pMLU; i.e., phonological complexity based on the accuracy in producing consonants, diphthongs, and complex syllabic structure) and phonological whole-word proximity to the corresponding adult word-form (i.e., proximity) scores were obtained for complexity and closeness to the target productions, respectively (Ingram, 2002). The pMLU of the word list from this tool is 8.31. This would be the value obtained if all target words were correctly produced, the proximity score in this case (child’s pMLU/target pMLU) being 1. Analysis of the original data from a sample (*N* = 50) of four-year-old children from Bosch (2004), used as a reference group, yielded the following values: mean pMLU = 7.55 (SD = 0.55) and mean proximity score = 0.91 (SD = 0.064).

Expressive language complexity was assessed from recordings of spontaneous utterances produced in a semistructured conversation/play situation using toys and picture books. The children's spontaneous utterances were transcribed so that the values of the MLU in words and the MLU of the five longest utterances (max-L) could be obtained. These measures could then be compared to the reference values from an age-matched control group of *N* = 50 Spanish-speaking children at four years old (Vázquez and Alonso, 2007). Mean MLU in words for this reference group is 3.91 (±1 SD: 4.45–3.37). Mean value of the max-L score is 10.56 (±1 SD: 12.47–8.65).

### Statistical analysis

At the individual level, the data from each patient were compared with the level of performance of an age-matched control group. The comparisons were performed using the modified *t* test, which is specifically designed to compare an individual to a small sample of control participants (Crawford et al., 2010).

At the group level, Mann–Whitney tests for independent samples were used to compare the behavioral scores of children with stroke to those of a control group when present (i.e., phonological measures of complexity and proximity, pMLU, and utterance complexity in spontaneous speech, MLU and L-max).

### MRI procedure

#### Sedation procedure

We followed exactly the same procedure as in a previous paper (François et al., 2016). Specifically, to reduce movement artifact of the participant during the MRI examination, the children were previously sedated as recommended in pediatric functional neuroimaging (Souweidane et al., 1999; Kerssens et al., 2005; Lai et al., 2011; Bernal et al., 2012; DiFrancesco et al., 2013). The children were evaluated by the anesthesiologist and food intake restriction was controlled. First, and with the aim of acquiring structural MRI information (structural MRI and DW-MRI sequences require precise and careful control of infant’s head and body movements), anesthesia was induced via a mask with sevoﬂurane which is the routine method used in François et al. (2016). Immediately after this procedure, the intravenous line was placed, and the children were transitioned to an intravenous-based anesthetic with propofol. The initial propofol dose was adjusted to render the children motionless but able to maintain his or her airway with a laryngeal mask. Propofol dosage for induction was 2 mg/kg and after perfusion, a dosage of 8–10 mg/kg/h was administered. The dosage was decreased to 6 mg/kg/h until the end of the procedure. Both sevoflurane and propofol have very small side effects and are drugs routinely used in pediatric neuroimaging. Duration of sedation using both drugs is very small, allowing for very fast recovery times (Bernal et al., 2012). Importantly, we considered the pharmacokinetics of sevoflurane to ensure that functional tasks were carried exclusively under propofol anesthesia. Because of this, we induced a fast transition to propofol anesthesia after sevoﬂurane and also, we ran the first 18 min of the structural imaging part ensuring that the possible effect of sevoflurane in the subsequent fMRI task was minimum. Indeed, the low solubility of sevoflurane in the blood induces a rapidly decreasing alveolar concentration after cessation of the inhaled agent which is linked to very fast recovery times and wash out (Yasuda et al., 1991; Bernal et al., 2012). However, and given that propofol can reduce connectivity in cortical areas of significance for the current study (Boveroux et al., 2010), we reconstructed standard resting-state fMRI (rs-fMRI) networks [i.e., the default mode network (DMN)] using independent component analysis (ICA) as a demonstration of data quality. Pre-processed resting state data from all patients (see below fMRI preprocessing and statistical analysis; data were normalized to a four-year-old template space) were submitted to Group Spatial ICA, using the Group ICA of fMRI Toolbox (GIFT v4.0b; http://mialab.mrn.org/software/gift/; Calhoun et al., 2001a). The mean value per time point was removed and data were concatenated and reduced to 10 independent components using a two-step principal component analysis and the infomax algorithm (Bell and Sejnowski, 1995). Finally, data were scaled to z-scores. To obtain whole brain group-wise statistics, the spatial map of each of the ICA components (i.e., networks) retrieved for all participants was submitted to a second-level analysis using a one sample *t* test under SPM12, which treats each subject’s spatial map as a random effect (Calhoun et al., 2001b). The DMN was selected by visually inspecting this group results. This group DMN along with each patient´s individual DMN are shown in Figure 1*B*.

### MRI scanning

MRI data were collected on a 1.5 T whole-body MRI scanner at a location which will be identified if the article is published (GE Signa HD). The acquisition of a high-resolution T1-w structural image (magnetization-prepared rapid acquisition gradient echo sequence; TR = 12.396 ms, TE = 5.22 ms, slice thickness = 0.4297 mm, 1-mm in-plane resolution, 180 slices, matrix size = 512 × 512) was followed by a DW-MRI sequence. DW-MRI scans were acquired with a spin-echo echoplanar imaging (EPI) sequence (TR = 16500 ms, TE = 100 ms, 48 axial slices, slice thickness 2.5 mm, FOV: 270, acquisition matrix: 100 × 100, reconstruction matrix: 256 × 256, voxel size: 1.05 × 1.05 × 2.5 mm^3^). One run with one non-diffusion weighted volume (using a spin-echo EPI sequence coverage of the whole head) and 29 diffusion-weighted volumes (b value of 1500 s/mm^2^) was acquired. One final FLAIR image (TR = 11002 ms, TE = 95 ms, slice thickness = 0.3711 mm, 4.5-mm in-plane resolution, 35 coronal slices, matrix size = 512 × 512) was also collected. After structural data were collected, a fMRI language task was conducted. One functional run consisting of 120 (6 min) functional images sensitive to BOLD contrast [echoplanar T2*-weighted gradient echo sequence; TR = 3000 ms (as in Perani et al., 2010, 2011) TE = 60 ms, flip angle 80°, acquisition matrix = 64 × 64, 3.75-mm in-plane resolution, 3.5-mm thickness, no gap, 34 axial slices aligned to the plane intersecting the anterior and posterior commissures] was acquired. Additionally, 100 volumes (5 min) of resting state (no stimuli were presented during acquisition) were collected using the same acquisition parameters as for the passive listening task.

DW-MRI data for the control group were part of the expanded diffusion tensor imaging release 5.1 of the NIH Pediatric MRI Data Repository (five controls: Siemens, 10 volumes with b = 0 s/mm^2^ and 50 directions with a b value of 1100 s/mm^2^, TR: 7900 ms, TE: 87 ms, 60 axial slices, FOV: 240, acquisition matrix: 96 × 96, voxel size: 2.5 × 2.5 × 2.5 mm^3^; one control with same parameters as the previous, but with 7 b0s and 46 directions; one control with same parameters as the first five controls, but with TR: 7800 ms and TE:85; one control with same parameters as the first five controls, but with TR: 8700 ms and 66 axial slices; one control, General Electric, Siemens, 2 volumes with b = 0 s/mm^2^ and 15 directions with a b value of 1100 s/mm^2^, TR: 19580.5 ms, TE: 84.8 ms, 60 axial slices, FOV: 240, acquisition matrix: 9 × 96, voxel size: 1.88 × 1.88 × 2.5 mm^3^).

### fMRI experimental design

For the language task, we used a spontaneous narration of a short children story (*The Snowman* by Raymond Briggs) recorded in child-directed speech by a female native Spanish speaker. The story was divided into ten blocks of 20 s, each block containing short sentences forming a sequence of complete intonation phrases.

As an acoustic control for the language task, we generated music-like stimuli composed of sequences of discrete tones using the technique developed in (Patel et al., 1998; see also Liu et al., 2010) for converting intonation patterns of spoken utterances to tone analogs. For each language sentence, a tone sequence was synthesized with the same pitch and temporal patterns as for the sentence’s syllables. First, discrete tone analogs were generated for each syllable in every sentence using a specific algorithm. This new sound represented the sum of the fundamental frequency at the median F0 of the original syllable [= (max F0 + min F0)/2] plus its seven odd harmonics (of the same amplitude and with sine phase) and was sampled at 44.1 kHz. Importantly, the tone analog had the same duration as the original syllable, to equate syllable duration. An 8-ms onset and offset taper was later applied to the tone to adjust the rise/decay time. The tone analogs of all the syllables and in each sentence were combined together, preserving the silent gaps as in the originally spoken utterances, to form a discrete-tone sequence. Using this technique, we ensured that each speech sentence had a musical-phrase correspondence comparable in overall parameters such as length, rate as well as in more ﬁne-grained patterns of frequency and timing.

Ten blocks of 20 s containing the original story (language condition) or its musical analog (control condition) were presented in order, with 15 s rest periods between blocks where no stimuli were presented (rest condition). The off-resting periods were set to 15 s because the BOLD response in children returns to baseline levels faster than in adults (Richter and Richter, 2003; Blasi et al., 2011).

### fMRI preprocessing and statistical analysis

Data were pre-processed using Statistical Parameter Mapping software (SPM8, Wellcome Trust Center for Neuroimaging, University College, London, United Kingdom; www.fil.ion.ucl.ac.uk/spm/). The functional images (language task and the rs-fMRI data) was first realigned, and then co-registered to the corresponding T1 using FSL’s FLIRT (FMRIB’s Linear Image Registration Tool; Jenkinson et al., 2012). As stated before, we used an age-appropriate template to register the children MRI images to the same space (Richards et al., 2016). In particular, we employed FSL’s FNIRT (FMRIB’s Nonlinear Image Registration Tool; Jenkinson et al., 2012), to normalize our patient’s T1-w and its corresponding lesion mask to a four-year-old MRI brain template obtained from the Neurodevelopmental MRI Database (https://jerlab.sc.edu/projects/neurodevelopmental-mri-database/; Almli et al., 2007; Sanchez et al., 2012; Fillmore et al., 2015; Richards et al., 2016). We first linearly registered the individuals’ T1-w and the corresponding lesion map to the four-year-old template using FLIRT. Then, we used FNIRT to non-linearly register the children T1 images to the aforementioned age-specific brain template. For all these steps, cost function masking was employed (Brett et al., 2001; Andersen et al., 2010; Ripollés et al., 2012). The registration parameters obtained during these calculations were applied to the lesion masks, the fMRI and the rs-fMRI data (previously registered to the corresponding T1) to normalize it to the common space (the four-year-old template). Finally, fMRI and rs-fMRI data were spatially smoothed with an 8-mm FWHM kernel.

Finally, an fMRI group analysis was performed. First, a blocked-design matrix was specified using the canonical hemodynamic response function. Three different conditions were specified: language, control, and rest. Data were high-pass filtered (to a maximum of 1/128 Hz), and serial autocorrelations were estimated using an autoregressive [AR(1)] model. Remaining motion effects were minimized by also including the estimated movement parameters in the model. A language > rest and control > rest contrast was calculated. These two contrasts were entered into a second-level paired samples model in which the language > control contrast was calculated for the left hemispheric damage group. Only clusters surviving an uncorrected threshold of *p* < 0.001 at the voxel level with a minimum cluster size of five voxels are reported.

### Gray matter (GM) and WM loss and precise lesion location

To precisely locate the areas affected by the lesion in each patient, we used the four-year-old adapted LONI LPBA40 brain atlas included in the Neurodevelopmental MRI Database (Shattuck et al., 2008; Richards et al., 2016) and the lesion masks that were registered to this space. In addition, to provide a measure of GM and WM loss, we computed an estimation of the lesion size (Tillema et al., 2008) as follows. We segmented the T1-w images into GM and WM using the four-year-old GM and WM segmentation priors included in the Neurodevelopmental MRI Database (Richards et al., 2016) using Unified Segmentation (Ashburner and Friston, 2005). The cerebellum and brainstem were then masked out using the manual four-year-old atlas of the Neurodevelopmental MRI Database (Fillmore et al., 2015; Richards et al., 2016). The ratios between the total GM and WM volume in both left and right hemispheres were computed and converted to a percentage of left cerebral hemisphere loss due to stroke (Tillema et al., 2008). Recent evidence shows that although there are differences between left and right GM/WM volumes in healthy controls, these should not exceed 1% (Carne et al., 2006).

### DW-MRI pre-processing

Diffusion data processing started by correcting for eddy current distortions and head motion using FMRIB’s Diffusion Toolbox (FDT), which is part of the FMRIB Software Library (FSL 5.0.1, www.fmrib.ox.ac.uk/fsl/; Jenkinson et al., 2012). Subsequently, the gradient matrix was rotated (Leemans and Jones 2009). Following this, brain extraction was performed using the Brain Extraction Tool (Smith et al., 2006), which is also part of the FSL distribution. The analysis continued with the reconstruction of the diffusion tensors using the linear least-squares algorithm included in Diffusion Toolkit 0.6.2.2 (Ruopeng Wang, Van J. Wedeen, TrackVis.org/dtk, Martinos Center for Biomedical Imaging, Massachusetts General Hospital). This pipeline of analysis was chosen as our acquisition parameters where fine-tuned using this procedure (François et al., 2016).

For the control group, we directly used the tensors provided by the NIH Pediatric MRI Data Repository (already preprocessed using the standard TORTOISE pipeline; Pierpaoli et al., 2010). We chose to use the already processed tensors to use the data with the best quality provided by the repository. These tensors were transformed into a compatible format with Diffusion Toolkit using in-house scripts (the code is available at https://github.com/pripolles/Diffusion-Tools/).

For both patient and control data, whole-brain tractography was performed using Diffusion Toolkit 0.6.2.210 and the interpolated streamlines algorithm. Tractography was started only in voxels with an fractional anisotropy (FA) value >0.2 and was stopped when the angle between two consecutive steps was larger than 35°. Deterministic tracking was performed by means of a two region-of-interest (ROI) approach using the TrackVis software (www.trackvis.org; Ruopeng Wang, Van J. Wedeen, TrackVis.org, Martinos Center for Biomedical Imaging, Massachusetts General Hospital).

### Deterministic tractography

Given the nature of our dataset (pediatric individuals with lesions) we decided to use manual deterministic tractography to dissect the tracts of interest, as it allowed us to draw specific ROIs adapted to each individual. In other words, for each individual and tract we drawn in native space-specific ROIs that took into account the neuroanatomy of each participant.

For each dataset, the three segments of AF were defined following the procedure reported by Catani et al. (2007) and also used in López-Barroso et al. (2013), Tuomiranta et al. (2014), and François et al. (2016). All ROIs were defined using the FA or RGB FA map as a reference. These FA and RGB-FA maps were generated from diffusion tensors that were reconstructed using the linear least-squares method provided in the Diffusion Toolkit. The ROI for the frontal area was placed in the coronal plane, between the central fissure and the cortical projection of the tract. The ROI for the temporal area was placed in the axial plane encompassing the fibers descending to the posterior temporal lobe through the posterior portion of the temporal stem. The third 2D ROI was defined at the sagittal plane encompassing the angular and supramarginal gyri of the IPL. to virtually dissect the three segments of interest, different two-ROI combinations were applied. The streamlines going through the frontal and temporal ROIs were classified as the long segment of the AF, the streamlines connecting the temporal and parietal ROIs constituted the posterior segment of AF, and the streamlines passing through the frontal and parietal ROIs formed the anterior segment of the AF. This process was conducted for both the left and the right hemisphere. As a control, we also performed virtual *in vivo* dissections of the IFOF, ILF, and UF. These dissections were performed for both hemispheres. For each dataset, we placed three spherical ROIs (as in Ripollés et al., 2017) at the level of the anterior temporal lobe (temporal ROI), the posterior region located between the occipital and temporal lobe (occipital ROI) and the anterior floor of the external/extreme capsule (frontal ROI). to define each of the tracts of interest we applied, again, a two-ROI approach. The ILF was obtained by connecting the temporal and occipital ROIs. The streamlines passing through the occipital lobe and frontal ROIs were considered as part of the IFOF. The frontal capsule ROI was united to the temporal ROI to delineate the UF. All these ROIs were applied according to anatomic landmarks defined in the research report by Catani and Thiebaut de Schotten (2008). In addition, the exclusion of single fiber structures that do not represent part of the dissected tracts was achieved using subject-specific no-ROIs. This procedure was done for both patients and controls.

Given the location of the patients’ lesions, we analyzed the volume of the AF. The volume was chosen as recent investigations revealed that this WM parameter is sensitive to individual differences (Saygin et al., 2013; Ocklenburg et al., 2014; Sreedharan et al., 2015; Vaquero et al., 2017) and that is also related to word-learning (Assaneo et al., 2019). We extracted the volume from the sum of the three segments of each hemisphere to obtain the values for complete-left and complete-right AF. The lateralization index was calculated [lateralization index = (values on the R − values on the L)/(values on the R + values on the L)] and was included in the analysis to see whether right WM reorganization had a relation with language development. The lateralization index ranges from –1 to 1: negative values represent left lateralization, values around zero symmetrical distribution, and positive values right lateralization (López-Barroso et al., 2013). As a control, we also calculated the LI for the IFOF, ILF, and UF using the same procedure. We compared the LIs for the AF, ILF, IFOF, and UF between controls and patients using non-parametric Wilcoxon Mann–Whitney tests. Due to the limited number of patients, confirmatory Bayesian statistical analyses were computed for all these between group comparisons using JASP with default (Rouder and Morey, 2012; Morey and Rouder, 2015; JASP Team, 2018). We used Bayes factors (BF_10_), which for the comparison at hand, reflect how likely data are different (e.g., a BF_10_ = 3 implies that a difference between groups is three times more likely to be observed under the alternative hypothesis).

To explore the link between the volume of the AF and productive aspects of language, we performed four non-parametric Spearman’s correlations between the volume of the complete AF and (1) the two measures of phonological development (phonological complexity and proximity scores) and (2) the two measures of expressive language complexity (MLU and L-max). To correct for multiple comparisons, the false discovery rate (FDR) was controlled at δ = 0.05 where multiple tests are performed (Benjamini and Hochberg, 1995).

### rs-fMRI functional connectivity analysis

The rs-fMRI data were analyzed following the rationale of the tractography analyses. The already preprocessed rs-fMRI data were analyzed using the functional connectivity (CONN) toolbox v17f (Whitfield-Gabrieli and Nieto-Castanon, 2012). After preprocessing, the images were bandpass filtered to 0.008–0.09 Hz and denoised by regressing out the six motion parameters obtained during realignment and the white-matter and cerebrospinal fluid signals. Finally, data were detrended. Given the tractography results (see Results) which pointed to a crucial involvement of the arcuate fasciculus in language functions in the studied patient population, we performed an rs-fMRI analysis that further confirmed these results. With this aim, we selected the two main regions connected by the arcuate fasciculus: the superior temporal gyrus (STG) and the inferior frontal gyrus (IFG; including both part opercularis and triangularis). These ROIs were extracted from the four-year-old adapted LONI LPBA40 brain atlas included in the Neurodevelopmental MRI Database (Shattuck et al., 2008; Richards et al., 2016). Data for all voxels within an ROI were averaged and one correlation per participant and hemisphere was calculated between the STG and the IFG. These correlation coefficients were converted to z values using Fisher’s r-to-z transformation. Right hemispheric z values for each of the four correlations were subtracted from their left-hemispheric counterparts to obtain an rs-fMRI laterality index analog to the one obtained for tractography (for a similar analysis, see López-Barroso et al., 2013). Associations between these laterality indexes and (1) the two measures of phonological development (phonological complexity and proximity scores) and (2) the two measures of expressive language complexity (MLU and L-max) were then explored using Spearman’s correlations. To correct for multiple comparisons, the FDR was controlled at δ = 0.05 where multiple tests are performed (Benjamini and Hochberg, 1995).

## Results

### Neuropsychological testing

#### Peabody picture vocabulary test

**Figure 2.**
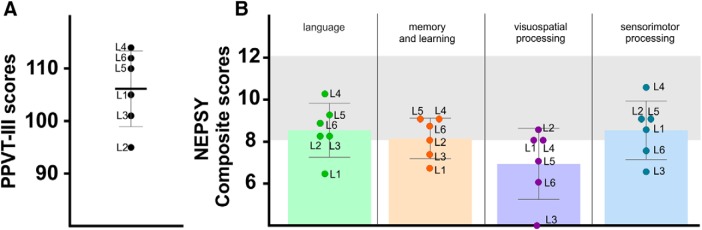
Cognitive outcomes. ***A***, Results of the PPVT-III test for each patient. The bars show the mean and SD. ***B***, Means and SDs barplots of the composite scores from the NEPSY-II. The gray slots indicate normative scores range (8–12).

The scores obtained in the Peabody vocabulary test (PPVT-III, with Spanish norms) yielded an estimate of verbal IQ which fells within normal limits with all the children having normal verbal IQ (Fig. 2*A*).

##### NEPSY II

**Figure 3.**
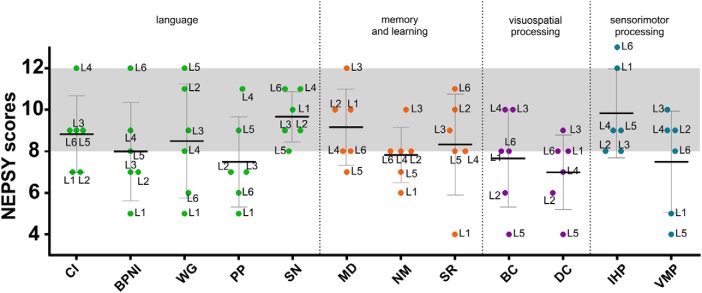
Means and SDs boxplots in the different subtests of the NEPSY-II (CI, comprehension of instruction; BPNI, body-part naming and identification; WG, word generation; PP, phonological processing; SN, speeded naming; MD, memory for designs; NM, narrative memory; SR, sentence repetition; BC, block construction; DC, design copying; IHP, imitating hand positions; VMP, visuomotor precision) grouped in domains (green, language; orange, memory and learning; purple, visuospatial processing; blue, sensorimotor processing). The gray slot indicates normative scores range (8–12).

The scores obtained from selected subtests of the NEPSY-II were first analyzed separately for each subtest. Composite scores corresponding to four of the specific cognitive domains of the NEPSY-II (i.e., language, memory and learning, visuospatial and sensorimotor processing; see Materials and Methods) were also computed by averaging the scalar scores obtained in the target subtests from each domain. As a group, the results showed that the mean scalar scores fell within the normal range (i.e., 8–12, 27th–75th percentile) except for phonological processing, narrative memory, block construction, design copying and visuomotor precision subtests showing mean scores slightly below the expected values (Fig. 3). When considering composite scores, the mean score in the language domain, although relatively low, fell within the normal range. Similar values were obtained in both the memory and learning and the sensorimotor domains. However, the mean composite score in the visuospatial processing domain was clearly below the normal range with a mean value <8, corresponding to 11th–25th percentile values.

##### Phonological development

**Figure 4.**
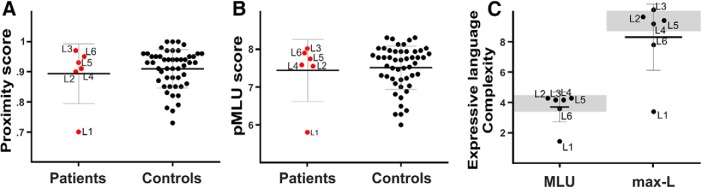
Expressive language measures. Means and SDs boxplots for children with PAIS and controls, for proximity and complexity scores. The graphs represent individual scores for the proximity score (***A***) for the pMLU scores (***B***) and from the referent group (*N* = 50), the group mean value (in black) and the score from each patient (in red). Maximum score for the pMLU score (complexity of the segmental structure of the words (consonants) is 8.31; maximum score for proximity score is 1. ***C***, Utterance complexity in spontaneous speech with the MLU in words and the MLU of the five longest utterances (max-L) for each patient. The gray slots indicate the scores range from an age-matched control group of *N* = 50 Spanish-speaking children at age 4 (Vázquez and Alonso, 2007).

Two measures, phonological complexity (phonological MLU, pMLU) and phonological whole-word proximity (proximity score) were obtained. We first compared the individual performance to an age-matched control group using the modified *t* test which is specifically designed to compare a patient to a small sample of control participants (Crawford et al., 2010). At the individual level, only one child (L1) presented significantly lower complexity score than controls (*p* = 0.002, modified *t* test, one-tailed). This patient presented a particularly poor level of performance in the measure of phonological proximity (*p* < 0.001, modified *t* test, one-tailed; Table 2; Figs. 4*A*,*B*). At the group level, the Mann–Whitney tests comparing children with PAIS to controls did not reveal significant differences (*p* > 0.05).

**Table 2.** Phonological complexity and proximity scores for the children with PAIS (single values) and for the control group (means and SDs)

	Phonological complexity	Phonological proximity
L1	5.81*	0.70*
L2	7.56	0.90
L3	8.03	0.97
L4	7.59	0.91
L5	7.75	0.93
L6	7.91	0.95
Controls	7.44 (0.81)	0.89 (0.09)

An asterisk indicates scores significantly different from the control group (*p* < 0.05) resulted from the modified *t* test.

#### Utterance complexity in spontaneous speech

To explore productive language with a specific focus on syntactic complexity, we evaluated the children expressive language complexity by using the MLU in words and the mean length of the five longest utterances (max-L). At the individual level, L1 presented a particularly poor performance in both MLU and max-L scores (*p* < 0.001, modified *t* test, one-tailed; Table 3; Fig. 4*C*).

**Table 3.** Spontaneous utterance performance (MLU and L-max scores) for the children with PAIS and for the control group (means and SDs)

	MLU	L-max
L1	1.74*	4*
L2	4.2	9.4
L3	4.1	9.8
L4	4.1	9
L5	4.2	9.2
L6	3.6	7.8
Controls	3.66 (0.96)	8.2 (2.16)

An asterisk indicates scores significantly different from the control group (*p* < 0.05) resulted from the modified *t* test.

### Neuroimaging results

#### Lesion location

The lesions affected different left perisylvian language-related areas (including the IFG, the middle temporal gyrus, the STG and the precentral gyrus; Table 4; Fig. 1). The analysis of the extent of the lesions reflected by the percentage of left cerebral hemisphere loss due to stroke revealed that WM volume loss was higher than that of GM in four out of six children suggesting that the lesions affected mainly WM tissue.

**Table 4.** Precise lesion location for each child with PAIS with the corresponding percentage over the total of the individual lesion size

		% of GM loss		% of WM loss	
Patient code	Lesion location	Left	Right	Left	Right
L1	L precentral gyrus (39%) L IFG (19%) L postcentral gyrus (16%) L MFG (16%)	0.37	.	2.5	.
L2	L SMG (56%) L AG (33%) L STG (10%)	4.4	.	7.7	.
L3	L AG (40%) L MOG (25%) L SMG (12%)	7.8	.	10.3	.
L4	L postcentral gyrus (53%) L SMG (23%) L precentral gyrus (20%)	4.3	.	0.8	.
L5	L AG (28%) L STG (26%) L SMG (19%) L MTG (8%)	12.1	.	7.4	.
L6	L IFG (39%) L MFG (33%) L precentral gyrus (24%)	0.6	.	1.7	.

The percentage of WM and GM loss is also presented for each patient. MFG, middle frontal gyrus; SMG, supramarginal gyrus; AG, angular gyrus; MTG, middle temporal gyrus; MOG, middle occipital gyrus.

#### fMRI task

**Figure 5.**
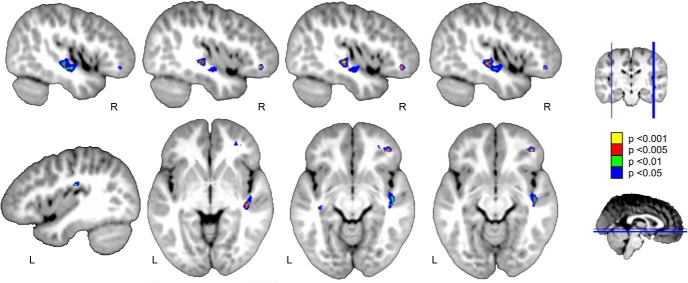
fMRI activation in the passive listening task for the language versus control contrast in patients. Two significant clusters with enhanced fMRI activity are found over the right inferior frontal and middle temporal gyri. Color-coded results are shown in yellow and at a *p* < 0.001, in red at *p* < 0.005, in green at *p* < 0.01, and in blue at *p* < 0.05 uncorrected threshold with five voxels of cluster extent, over the Neurodevelopmental MRI Database four-year-old template from Richards et al. (2016). Neurologic convention is used.

We obtained the functional activations from all the children using a passive story-telling task during which a short children’s story was auditorily presented in 20-s-long blocks (language condition). A control condition, consisting of an acoustically-matched set of music-like stimuli, was also presented (see Materials and Methods); the goal of this contrast was to elicit activity related more specifically to linguistic features of the stimuli (especially phonological and semantic), rather than prosodic or more low-level auditory cues (such as duration or loudness) which were similar for the two stimulus types. The language versus the acoustic control condition contrast yielded significant (but uncorrected) activity in the right middle temporal gyrus and in the right IFG (*p* < 0.001 uncorrected; Fig. 5). No consistent activity pattern was observed in the reverse contrast (music-like condition vs language). Note, however, that due the limited *N*, these results are not corrected for multiple comparisons.

#### Deterministic tractography

**Figure 6.**
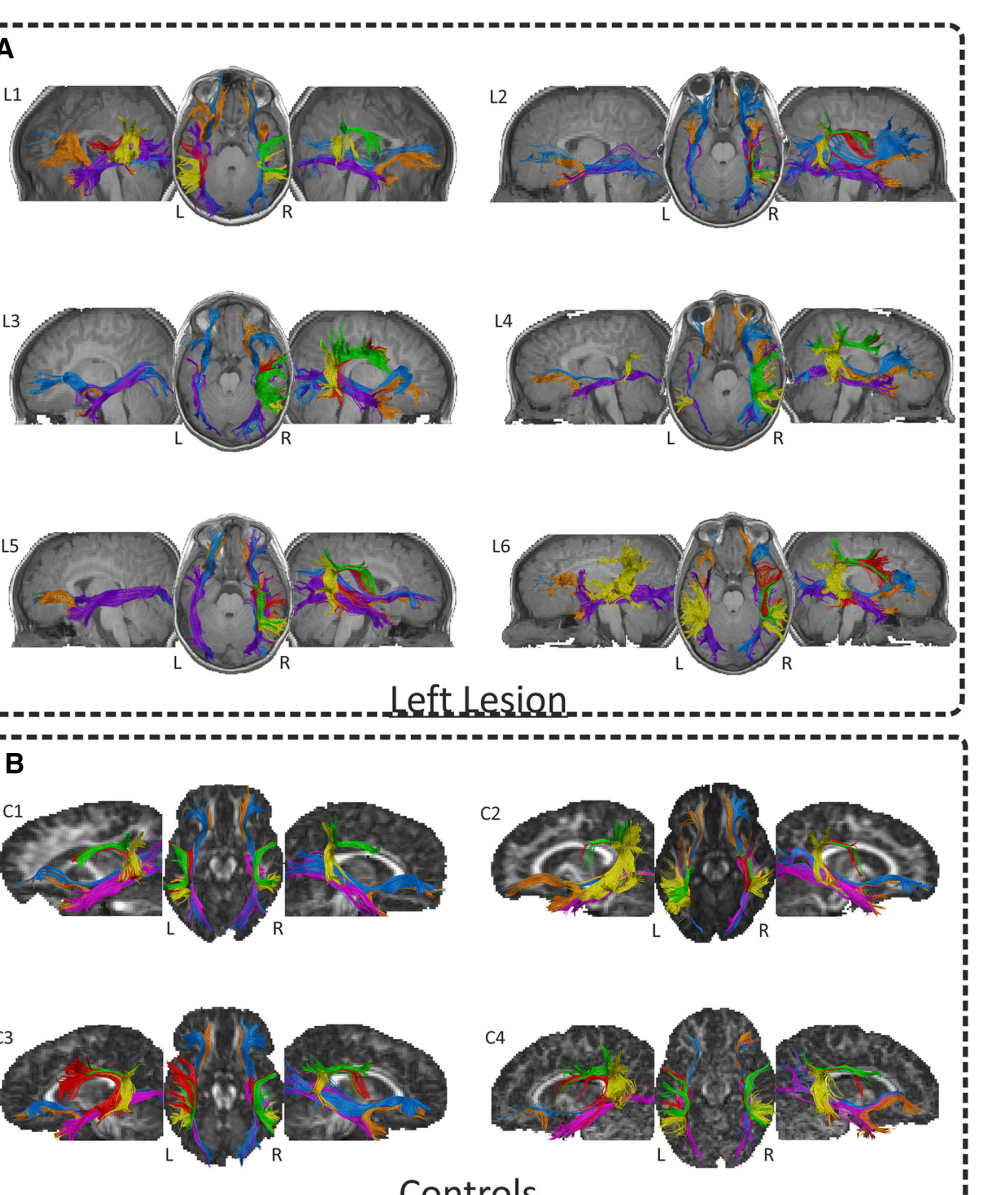
Deterministic tractography. DTI *in vivo* dissections of the four tracts of interest AF/ILF/UF/IFOF shown in native space on the T1-w images in each patient (***A***) and four controls (***B***). Neurologic convention is used. The two-ROI approach revealed that for all the children with a left PAIS (***A***), the three segments of the AF were well preserved in the right hemisphere. Meanwhile, we were not able to dissect the left AF in most of them (L4 and L6 presented the posterior segment only, whereas L1 presented the posterior and the long segment but not the anterior one). We did not observe any substantial damage for the ventral WM pathways (ILF, IFOF, and UF) in either hemisphere. By contrast, the three segments of the left AF were always present in control children (***B***). However, while the anterior and posterior segments of the right AF were always present in these children, we were not able to reconstruct the long segment of the AF in three controls.

The deterministic *in vivo* dissections using the two-ROI approach revealed that for all the children with PAIS, the three segments of the AF were well preserved in the right hemisphere. Meanwhile, we were not able to dissect the left AF in most of them (L4 and L6 presented the posterior segment only, whereas L1 presented the posterior and the long segment but not the anterior one). We did not observe any substantial damage to the ventral WM pathways (ILF, IFOF, and UF) in either hemisphere (Fig. 6).

Deterministic dissections in the control group revealed that the three segments of the left AF were always present. By contrast, while the anterior and posterior segments of the right AF were always present, we were not able to reconstruct the long segment of the AF in three control children. Crucially, when comparing the laterality index for the volume of the complete AF in patients and controls we found that patients presented a significantly more right-lateralized AF than controls (*U* = 0; *p* < 0.001; BF_10_ = 147.79). Importantly, when comparing the volume of the left and of the right AF between patients and controls, we observed significantly reduced volume of the left AF in patients (*U* = 5; *p* < 0.009; BF_10_ = 36.95) and a larger volume of the right AF (*U* = 6; *p* < 0.013; BF_10_ = 6.73) in patients as compared to controls. Moreover, to rule out possible group-specific differences due to the different scanning parameters and procedures used between groups, we also compared the laterality indexes of several control tracts also related to language. We found no differences between the laterality indexes of patients and controls for the ILF (*U* = 18; *p* = 0.289; BF_10_ = 0.607), IFOF (*U* = 25, *p* = 0.814; BF_10_ = 0.473), or UF (*U* = 20; *p* = 0.409; BF_10_ = 0.574), which further proves that our results were specific to the AF.

#### Brain-behavior relationships

Given that the dorsal pathway was the most affected in patients (the laterality of the entire AF was significantly more right-lateralized in patients than in controls), we computed the laterality indices for both dorsal structural and functional data (see Materials and Methods). Due to the limited number of patients, confirmatory Bayesian statistical analyses were computed for all the brain-behavior correlations with the software JASP using default priors and the Bayesian correlation module (using Kendall’s τ-b; JASP Team, 2018; Morey and Rouder, 2015; Rouder and Morey, 2012). We used Bayes factors (BF_10_), which for the comparison at hand, reflect how likely data are correlated (e.g., a BF_10_ = 3 implies that a positive correlation is three times more likely to be observed under the alternative hypothesis).

**Figure 7.**
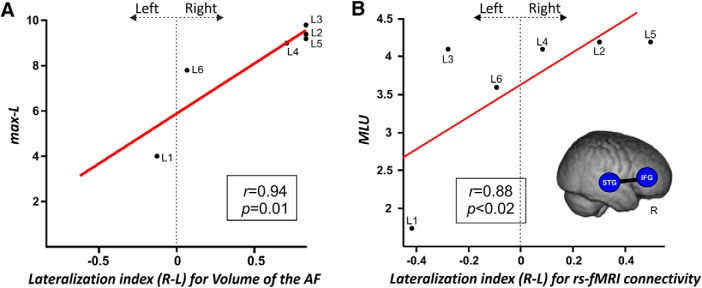
***A***, Correlation between DTI properties extracted from the manual reconstruction of the arcuate fasciculus bilaterally and productive aspects of language. The positive correlation between the lateralization index for the volume of the complete right AF and the maximum length of utterance (L-max) is shown. Lateralization index: values closer to –1 mean lateralization to the left, values around 0 represent a symmetrical distribution, values closer to 1 mean lateralization to the right. This result evidences that the more lateralized to the right is the integrity of the AF, the better the productive aspects of language. ***B***, Correlation between rs-fMRI laterality indexes and productive aspects of language. The scatterplot shows the positive Spearman’s correlation between the lateralization index for the STG-IFG laterality index and the MLU. This result evidences that the more lateralized to the right the intrinsic connectivity between the regions connected by the AF, the better the productive aspects of language. A 3D render of the four-year-old template is displayed with the ROIs used in this analysis. Correlation index and *p* values are displayed.

We found a significant positive correlation (*r_s_*[6] = 0.94; *p* < 0.0001, FDR-corrected for multiple comparisons; BF_10_ = 5.876) between productive aspects of language (as measured by the max-L) and the lateralization index for the volume of the complete AF (Fig. 7*A*). This result indicated that the greater the reorganization was over the right hemisphere (i.e., higher lateralization index of AF’s volume), the better productive aspects of language (higher max-L values).

Following the tractography brain-behavior results, the rs-fMRI analyses showed that the laterality values for the intrinsic connectivity strength between the STG and IFG were positively associated with productive aspects of language (*r_s_*[6] = 0.88; *p* < 0.02; BF_10_ = 3.43) as measured by the MLU in spontaneous speech; Fig. 7*B*), although these analyses did not survive the FDR correction. Nonetheless, this finding suggests that the greater the reorganization was to the right hemisphere (i.e., the greater the intrinsic functional connectivity between these two regions in the right hemisphere), the better the productive aspects of language. This pattern of results strikingly parallels the one found for the lateralization of the AF using deterministic tractography and provides converging evidence that greater right hemispheric engagement was related to better productive aspects of language in this group of children.

## Discussion

In the present study, structural connectivity measures pointed toward an association between the right reorganization of the dorsal pathway and measures of expressive language development: the structural laterality indices of the right dorsal pathway predicted language production outcomes. Crucially, the structural laterality indices of the AF were significantly more right-lateralized in children with left PAIS than in a matched group of controls, while no differences were observed for the UF, ILF, or IFOF.

Although all the children had normal receptive vocabulary levels and verbal IQ, they presented slightly low language production and processing outcomes as a group. The finest measure of utterance complexity in spontaneous speech (L-max) showed that half of the children presented a limited syntactical development as compared to controls. The individual results from the NEPSY-II language subtests showed that most of the children were below normative scores except for the speeded naming subtest. Besides this, most of them had below normative scores in the visuospatial processing subtests. The latter result is in line with two studies reporting impaired visuospatial processing induced by left lesions (Lidzba et al., 2006a,b). Taken together, the data may indicate that the reorganization of the language network toward the right hemisphere can, in turn, impair functions originally supported by the right hemisphere, thus providing support to the so-called “crowding effect” induced by early stroke (Satz et al., 1994).

The fMRI results, although not corrected for multiple comparisons, revealed two clusters of activation over the contralesional hemisphere only, one in the right middle temporal gyrus and the other in the right IFG. Prosodic and voice processing have been recently shown to involve a dual processing stream organization with both a dorsal and a ventral pathway connecting the right posterior STG to the right IFG and premotor cortex (Pernet et al., 2015; Sammler et al., 2015; Zäske et al., 2017). Here, we used fine-grained control stimuli such that each speech sentence from the language condition had a musical-phrase correspondence with similar prosodic and acoustic features in the control condition. Therefore, these two clusters are not likely to reflect prosodic processing. Previous studies in healthy young adults have largely reported activations over these regions during active syntactic and semantic processing but with a clear left lateralization (Binder and Desai, 2011). Here, consistently with previous reports on language comprehension in children and adults with stroke (Jacola et al., 2006; François et al., 2016; Lidzba et al., 2017a,b), no activation over the left hemisphere was observed. Although these results must be taken with caution (due to the small *N*, the presence of anesthesia, the fMRI data were not corrected for multiple comparisons), our results may suggest that both the processing of acoustical features of the voice signal, as well as high-order syntactic processing, were taking place in our group of children.

Critically, both rs-fMRI and especially DW-MRI data provided converging evidence showing that the right hemisphere can take over language functions. Successful recovery after early stroke can be associated with both intra-hemispheric perilesional and interhemispheric contralesional plasticity mechanisms (Raja-Beharelle et al., 2010; Dick et al., 2013). However, it is important to bear in mind that different physiologic mechanisms of recovery may be triggered after arterial ischemic stroke or periventricular brain lesions which may, in turn, partly explain the different patterns of results reported here and in the study from Raja-Beharelle et al. (2010). Here, we found that compared to healthy controls, patients presented a smaller volume of the left AF together with a larger volume of its right homolog. These differences were transferred to the laterality index of the AF: the volume of the arcuate was more right-lateralized in patients than in controls. Interestingly, we found a clear positive association between the volume of the right AF and a measure of language production. Besides, we found an association between the laterality index of the intrinsic connectivity strength between perisylvian regions of the dorsal pathway and the MLU (although this result was not corrected for multiple comparisons). Taken together, these results indicate that reorganization of the language network over the right hemisphere can be accomplished through structural and functional hyperconnectivity between right temporal and frontal areas. An increased GM density in the right temporoparietal regions has been associated with a right-lateralized language network in a large group of children with drug-resistant left-sided lesional focal epilepsy (Pahs et al., 2013). The authors suggested that the size of the unaffected planum temporale may present a “reserve capacity” for interhemispheric language reorganization in the presence of lesions within left perisylvian regions. Additional evidence for the important role of plasticity of the right temporal cortex in the recovery of language functions came from studies conducted in left hemisphere stroke survivors with aphasia (Forkel et al., 2014; Xing et al., 2016; Hope et al., 2017; Lukic et al., 2017; Piai et al., 2017). A very recent study from Northam et al. (2018) gives more support to this hypothesis by showing that brain reorganization toward the right hemisphere can compensate production deficits often observed in children with left PAIS. Moreover, recent evidence exists in rats and humans showing that early lesions in the motor system can induce contralateral axonal sprouting (Liu et al., 2008; Schaechter et al., 2009). We speculate that the early disconnection of the left dorsal pathway may induce the degeneration of neurons projecting from temporal to frontal areas while triggering contralateral axonal sprouting over the right hemisphere. This contralateral reorganization mechanism may, in turn, foster contralateral increased GM plasticity changes reflected by structural and functional hyperconnectivity.

The present findings provide important results allowing the refinement of a recent model describing the emergence of increasingly complex linguistic functions through development (Catani and Bambini, 2014). Building on previous neuroanatomical models of language processing (Friederici 2011, 2012; Catani et al., 2005; Hickok and Poeppel, 2007; Rauschecker and Scott, 2009), this model links the stepwise evolution of language functions to the neuro-anatomic changes occurring during early development with a specific role of the posterior and long segments of the AF, necessary for normal language development and especially in word-learning, syntactic processing and verbal working memory (Lebel and Beaulieu, 2009; López-Barroso et al., 2013; Catani and Bambini, 2014; Budisavljevic et al., 2015). Nonetheless, these models remained elusive on the role of the right dorsal and ventral pathways. Prosodic cues are preferentially processed by the right hemisphere (Zatorre and Belin, 2001; Giraud et al., 2007; Perani et al., 2010) and are important for language acquisition as they may help bootstrapping language learning processes (Shukla et al., 2011; Gervain and Werker 2013; De Diego-Balaguer et al., 2015; Filippi et al., 2017; François et al., 2017). It has also been revealed that temporoparietal brain regions presented a slower maturation as compared to frontal regions in healthy infants (Leroy et al., 2011). This slow cortical maturation is paralleled by the slow maturation and high sensitivity to environmental factors of the right posterior segment of the AF (Budisavljevic et al., 2015). Our results, taken together with the previous evidence on the role of the right dorsal and ventral pathways for language processing, suggest that the right hemisphere may also play an important role in children’s development and in language processing after stroke. Besides, although still under debate, recent data from children with early acquired brain lesions show that the time-window for the compensatory recruitment of homologous right cortical regions after left stroke may already close at the age of five years (Lidzba et al., 2017a). Similarly to what has been observed in adults with aphasia (Forkel et al., 2014; Hartwigsen and Saur, 2019), the right temporoparietal regions and the posterior segment of the right AF may be critical for compensating the devastating effect of stroke. Therefore, the enhanced involvement of the right dorsal pathway after early stroke may induce functional and structural changes within a more flexible language network than lesions acquired in a highly specialized functional network during adulthood (Levine et al., 2016). Future research is needed to specify the role of genetic predispositions contributing to the left hemisphere specialization as opposed to the neuroplastic mechanisms occurring over the right hemisphere in the face of early left lesions.

The present study presents some limitations. The sample size is small, which is an intrinsic and recurrent problem in studying rare neonatal conditions. This limitation is particularly evident for the fMRI results which were not corrected for multiple comparisons. In addition, the fMRI task used here is novel in this type of population and results from a control group of healthy children and/or a group of adults without sedation is needed to further interpret our fMRI data in terms of functional reorganization. The presence of degeneration or necrosis may alter the microstructure of perilesional tissue, leading to problems in the estimation of fiber orientations, especially in areas with presence of crossing fibers, which can, in turn, induce artefacts in track reconstructions (Ciccarelli et al., 2006; Schonberg et al., 2006; Møller et al., 2007; Dell'Acqua, et al., 2010). The use of light anesthesia to avoid movements during the MRI acquisition may have influenced the functional activations as the rate of failure can be as high as 21% with propofol and an auditory task (Bernal et al., 2012; note, however, that we were able to reconstruct canonical ICA networks for each patient; Fig. 1*B*). The DWI data from the NIH database were acquired with different scanning parameters which may impact the comparison with the data obtained in our group patients. However, we think that this confound is unlikely to be affecting our results, since the differences observed were specific to the AF and not to the ventral tracts we used as a control. In addition, our sample was studied at around four years of age, and therefore is not yet fully developed (neither in terms of language abilities nor in terms of brain structure/function). Other language-related deficits could appear at later stages and the deficits shown here could also resolve by school age. Besides, this group of children was developing in a bilingual environment (five out of six). The type of language input received during the first years, such as the presence of co-speech gestures or the use of decontextualized talk, have been shown to play an important role in the development of linguistic functions after neonatal stroke specifically for those showing initial linguistic delays (Rowe et al., 2009; Demir et al., 2014, 2015). This might particularly be the case for the two patients (L2 and L6) who were not receiving speech therapy as previous studies have shown that interventions such as music-based therapy can induce functional and structural reorganization in children (Zipse et al., 2012). However, at present, the specific impact on bilingual experience in children with PAIS remains fairly unexplored. Taken together, these data suggest that the “catch-up” of linguistic functions previously reported once children reach elementary school (Bates, 1997) may already occur in preschoolers by four years of age, probably resulting from the enriched linguistic input provided by parents (Demir et al., 2014, 2015), caregivers and early speech stimulation programs.

In summary, we report the first 3D reconstructions of the dorsal and ventral language pathways together with fine-grained assessments of cognitive and language functions in a homogeneous group of four-year-old children who had a left PAIS. We provide evidence for an association between the structural and functional (although to a lesser extent) brain reorganization patterns toward the right hemisphere and language production in pre-school children with congenital lesions. We speculate that interhemispheric plasticity through structural and functional hyperconnectivity mechanisms might be crucial for positive outcomes following early lesions.

## Synthesis

Reviewing Editor: Sunghee Cho, Burke Neurological Institute/Feil Family Brain and Mind Research Institute, Weill Cornell Medicine Decisions are customarily a result of the Reviewing Editor and the peer reviewers coming together and discussing their recommendations until a consensus is reached. When revisions are invited, a fact-based synthesis statement explaining their decision and outlining what is needed to prepare a revision will be listed below. The following reviewer(s) agreed to reveal their identity: Melissa Thye.

The manuscript investigating structural and functional reorganization in children with perinatal ischemic stroke were evaluated by two reviewers who have expertise in the field. Both reviewers considered the manuscript very interesting and the findings from the study are a significant addition for the field. Despite some concern on the underpowered study design, it was acknowledged the challenge on recurring unique population of study subjects. Employing multimodal neuroimaging assessment and providing limitations in study design and interpretation were also noted. However, the reviewers raised several major points. These included insufficient introduction for readers on the neuroanatomical circuit for language processing, overinterpretation of findings that are not statistically supported, as well as some concern on length of manuscript- which requires a succinct description of highlights and main findings. An additional issue is the rationale on the inclusion of subjects with bilateral or right hemisphere lesion, as language impairment largely stems from left hemispheric lesion. Other detailed and specific comments from reviewers are below.

Reviewer 2

This manuscript describes an attempt to evaluate the neural reorganization that occurs after perinatal ischemic stroke and the association between the reorganization of critical language regions and white matter tracts and varied measures of receptive and productive language use. This study has a number of strengths, namely multimodal neuroimaging data from a unique population which provides an interesting avenue to study an experience-dependent hypothesis regarding neural reorganization during language development. Throughout the manuscript, the authors do a good job of articulating some of the limitations of their approach and data when interpreting their results, though I believe there are several additional issues that need to be considered.

Major points:

- The Introduction is underspecified in some areas. Specifically, there is very little information provided regarding the specific neuroanatomical model of language processing (e.g., which regions and white matter tracts are involved). For example, the authors state, “Although it is still under debate, some of the white-matter tracts supporting the language network are already developed and functional at birth, while others are still developing with a delayed maturation.” I understand the point the authors are highlighting with this claim, but I think at present, it's too vague and would be a more informative statement if the authors discussed specific white matter tracts (as the role of the arcuate fasciculus differs from the role of the uncinate fasciculus in language, for instance). Finally, the authors hypothesize that damage to left AF would result in the most language impairment which I think is a reasonable claim, but very little information has been provided concerning the role of AF in language processing.

- It is problematic to compare data from the patient group to data from healthy children collected using different scanning parameters and procedures. This creates a group-specific confound that impacts how the results are interpreted.

- Relatedly, additional information is needed to characterize the healthy control group:

- Was this sample also a bilingual sample? If not, would the differences in language acquisition and use between the experimental and control group impact the study and the inferences made at all?

- Were the healthy control participants also sedated following a similar procedure described for the patient group?

- Is there any available information detailing the language or cognitive functioning of the participants in the healthy control group?

- The diffusion data seems to have been processed using a different pipeline than the diffusion data from the experimental group. If this is the case, why not process all of the diffusion data using the same processing pipeline? Furthermore, was the other structural data processed using the pipeline applied to the experimental group?

- It is problematic to report and overly interpret results which have not been corrected for multiple comparisons - this occurs at several points throughout the manuscript. Also, was a cluster-level correction applied prior to reporting any of the fMRI results? Again, on page 17, the authors seem to interpret a finding that did not survive correction which is problematic.

- It is possible that I am misunderstanding the laterality index used in the manuscript, but the finding that children with LH lesions are more right lateralized than the healthy controls is somewhat misleading given the metric. For instance, if the metric is calculated by subtracting the LH values from the RH values and then dividing by the sum of values, the participants with the LH lesions would inevitably have ratios indicating right laterality. This laterality isn't necessary an indicator that they are recruiting homologous regions in the right hemisphere to a greater degree than the control participants - these groups could theoretically be recruiting the right hemisphere at a comparable level, but the ratio is being artificially inflated due to the lack of involvement of the left hemisphere. It's possible I'm missing a critical detail, but additional information would give me greater confidence in this comparison with the control group.

Minor points:

- The authors mention that two of the participants do not receive speech therapy - what impact might this have on the experience-dependent process being studied here?

- It appears that some of the diffusion data (and all of the diffusion data collected from the experimental group) were collected with non-isotropic voxels which can complicate tractography analyses - were any considerations made regarding the tractography analyses given this and given the varied parameters across participants?

- Is a TR of 3000 ms standard practice in infant neuroimaging studies - this seems like a long TR given, as the authors point out, that the BOLD response in children returns to baseline levels at a faster rate than in adults.

- This is a very minor point, but the authors mention on page 12, “These tensors were transformed into a compatible format with Diffusion Toolkit using in-house scripts.” In general, it might be better to provide more information regarding the details of an “in-house script” in order to increase the transparency of the method and allow other researchers to replicate your work. In this vein, I encourage (but am not requiring) the authors to use data and code-sharing practices like posting code on OSF and Gitlab/hub to promote open science.

Reviewer 3

This manuscript examines the pattern of brain functional and structural reorganization with regard to language outcomes in a group of nine 4-year-old children with perinatal arterial ischemic stroke. Children with only a left perinatal stroke (#6) showed larger lateralization indices of both functional and structural connectivity of the dorsal language pathway towards the right hemisphere. Three children had also right more than left hemisphere lesions. This functional and structural right lateralization was confirmed when the group of children with a left perinatal brain insult was compared to a group of matched healthy controls. Early lesions of the left dorsal pathway and associated perisylvian regions can induce the inter-hemispheric transfer of language functions to right homolog regions.

This is a very interesting study using various techniques in a challenging population. The findings are relevant to our understanding of how a brain might reorganize after a stroke, although recent published reports have shown similar effects. The manuscript is very wordy and at times difficult to read because it is so long. My first suggestion to the authors is to significantly reduce the manuscript in size to bring it down to a size that one can more easily read while at the same time highlighting the main findings. I think that the 3 subjects with either bilateral or right hemisphere lesions do not really contribute to this paper and do not resemble a group that is large enough to allow the authors to draw any meaningful conclusions.

More specific comments that the authors want to consider when they revise their manuscript.

1. The group of children is more heterogeneous than the paper makes us believe. In the adult world, heterogeneity is also defined by where the lesion is and what impact the lesion has on relevant structures (e.g., the AF-Lesion Load - Marchina et al. 2011 and Wang et al., 2013). In addition, the problem in his paper is that only six of the 9 subjects had left hemisphere lesions, two had bilateral lesions and one even had a right hemisphere lesion. Thus, there is already considerable individual heterogeneity due to differences in lesion size and location, now another variability is added which is “hemisphere affected”. I would recommend to exclude the children with bilateral or right hemisphere lesions from this report. In some parts of the results section, the authors contrast right hemisphere lesion (which is obviously only 1 out of 3 subjects) with left hemisphere lesions. I would recommend to drop this part entirely.

2. I would also want to see in the figures how the lesion interfered with the dorsal AF (see Wang or Marchina) or the functional connectivity between STG and IFG. This connectivity might be absent which could potentially explain the strong laterality.

3. How were these 6 or 9 children selected from the larger group of children with symptomatic PAIS? That would be important for understanding whether or not the findings here are generalizable.

4. It appears that the children were tested using a Spanish adaptation of the NEPSY-II battery, but the control group consists of those taken from a multi-site longitudinal study of typically developing children supported by the NIH, presumably in English speaking countries. That should be discussed.

5. Why was a deterministic tracking algorithm and not a probabilistic tracking used? When one does statistics on tract volume using deterministic tracking, then the size and location of the ROI used to do the tracking is very important. The paper does not clearly state whether or not the ROIs were drawn for each brain separately or if the same ROI template was used for all brains. Please specify. If it was drawn separately for each brain, then a measure of variability should be included and the authors should discuss what effect that might have on the volume of the AF.

6. fMRI: the language versus music condition contrast yielded significant clusters of activation in the right temporal and right IFG (but uncorrected). The authors say that this right hemisphere lateralization of language processing in children with early left-hemispheric damage might reflect functional reorganization. I would be a little bit more careful here. Since the two functional imaging tasks are new and novel in this patient population and since the patients are sedated, it would have been great to see the activation pattern in a control group of children without lesions or in a group of adults that was not sedated. The question is whether or not the right hemisphere pattern is due to the contrast of active vs control task, the left hemisphere lesion, or the sedation. If there is no control group, then this needs to be critically discussed.

7. The rs-fMRI pattern of lateralized connectivity largely reflects the structural lateralization using AF volume. Why was rsfMRI and DTI done? is there some additional information that rs-fMRI provides beyond the DTI information?

8. The authors seem to mainly use a lateralization index of the STG-IFG connectivity. How was the STG-IFG connectivity determined on the left hemisphere considering the lesions? Did the lesions involve the STG or IFG and how did this affect the functional connectivity?

9. The authors might be interested in a publication by Zipse et al., (2012) describing one subject of a monozygotic twin pair with a very large left hemisphere stroke and enormous development of the right AF in response to an intervention, but not of the right UF. A similar structural development was not seen in the other twin without any stroke, who was scanned multiple times over time
